# Immunogenecity of Modified Alkane Polymers Is Mediated through TLR1/2 Activation

**DOI:** 10.1371/journal.pone.0002438

**Published:** 2008-06-18

**Authors:** Radhashree Maitra, Cristina C. Clement, Giovanna M. Crisi, Neil Cobelli, Laura Santambrogio

**Affiliations:** 1 Department of Pathology, Albert Einstein College of Medicine, New York, New York, United States of America; 2 Department of Pathology, Baystate Medical Center, Springfield, Massachusetts, United States of America; 3 Division of Orthopedic Surgery, Montefiore Medical Center, New York, New York, United States of America; Instituto Oswaldo Cruz and FIOCRUZ, Brazil

## Abstract

**Background:**

With the advancement of biomedical technology, artificial materials have been developed to replace diseased, damaged or nonfunctional body parts. Among such materials, ultra high molecular weight alkane or modified alkyl polymers have been extensively used in heart valves, stents, pacemakers, ear implants, as well as total joint replacement devices. Although much research has been undertaken to design the most non-reactive biologically inert polyethylene derivatives, strong inflammatory responses followed by rejection and failure of the implant have been noted.

**Methodology/Principal Findings:**

Purification of the alkane polymers from the site of inflammation revealed extensive “in vivo” oxidation as detected by fourier transformed infra-red spectroscopy. Herein, we report the novel observation that oxidized alkane polymers induced activation of TLR1/2 pathway as determined by ligand dependent changes in intrinsic tyrosine fluorescence intensity and NF-κΒ luciferase gene assays. Oxidized polymers were very effective in activating dendritic cells and inducing secretion of pro-inflammatory cytokines. Molecular docking of the oxidized alkanes designated ligand specificity and polymeric conformations fitting into the TLR1/2 binding grooves.

**Conclusion/Significance:**

This is the first report of a synthetic polymer activating immune responses through TLR binding.

## Introduction

With the advancement of biomedical technology, artificial materials have been developed to replace diseased, damaged or nonfunctional body parts. Among such materials, ultra high molecular weight alkane or modified alkyl polymers (PE) have been extensively used in heart valves, stents, pacemakers, ear implants, as well as total joint replacement devices [1]. Based upon the most recent epidemiological data approximately three quarters of a million joint replacements were performed in the United States in 2005. Ten to fifteen percent of these joint replacements were classified as revision joint replacements due to implant failure. The single most common cause of failure and reason for revision is a process known as osteolysis [2]. Over time different size particles and short polymers of wear debris are generated from the PE implant [3]–[8]. These micron-size particles as well as low molecular weight alkane polymers are responsible for the initiation of an aseptic inflammatory response known as osteolysis [5], [9],[2]. The generation of implant-derived wear particles/polymers prompts an inflammatory reaction at the prosthesis-bone interface resulting in osteoclastic bone resorption around the implant and ultimately the mechanical failure of the device. The molecular mechanisms underlying these clinically important events are very poorly understood. There is a consensus in the literature that the size of the particles is important in determining the extent of the inflammatory process [10]. In particular it appears that smaller size particles (in the nanometer, micrometer range) which can be phagocyted by local and recruited antigen presenting cells are the most likely to cause the initial inflammation [10]. It has also been reported that macrophages and osteoclast upon phagocytosis of ultra high molecular weight polyethylene became activated and mounted a strong pro-inflammatory immune response mediated by TNF-a, IL1 and IL6 production [5]. However, the molecular basis for PE recognition by the immune system is not yet known.

## Results

**Figure 1:**
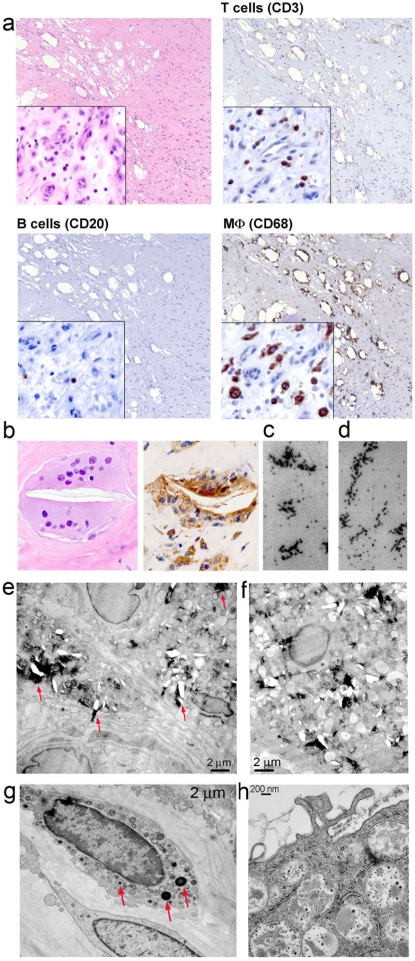
Periprosthetic inflammatory reaction to micron and sub-micron size modified n-alkane polymers. a) Immunohistochemistry of periprosthetic tissue retrieved at the time of the implant revision surgery (20X magnification and 40X for inset). The tissue was stained with: hematoxylin and eosin to detect total cell infiltrates, CD3 to detect T cells, CD20 to detect B cells and CD68 to detect macrophages. PE polymers of varied shapes and sizes are visualized as transparent areas. b) Hematoxylin and eosin staining (right) and CD68 (left) showing a multi-nucleated giant cell surrounding a large PE particle. c and d) Electron micrograph of sub micrometer PE particles c) purified from periprosthetic tissue or d) chemically synthesized mPE particles shown as control. e, f, g and h) Electron micrograph of periprosthetic tissue obtained from implant failure prosthesis. e and f) PE particles are visualized as black material indicated by red arrows. g and h) PE sub-micron particles, indicated by red arrows, phagocyted by local antigen presenting cells. A complete engorgement of the endocytic tract by post implant PE is observed in h.

Histological evaluation of periprosthetic implant associated tissue, retrieved from patients undergoing hip replacement surgery due to PE induced osteolysis, indicated chronic inflammatory infiltrates (Figure 1a). Immunophenotyping demonstrated that these infiltrates consisted predominantly of CD68^+^ macrophages (MΦ), with few T and B cell infiltrates (Figure 1a). Histiocytes and foreign body giant cells engulfing birefringent PE particles, generated by wear and tear of the polymeric implant, were also observed (Figure 1b).

Ultrastructural analysis revealed the presence of micron and sub-micron PE particles in the periprosthetic inflamed tissue (Figure 1c), similar to the one derived from chemically synthesized carbonyl modified alkane polymers (Figure 1d). Micron size PE particles were mostly extracellular and observed among collagen fibers (Figure 1e, 1f). Sub-micron PE particles on the other hand were phagocytosed by local antigen presenting cells (Figure 1g and 1h).

**Figure 2:**
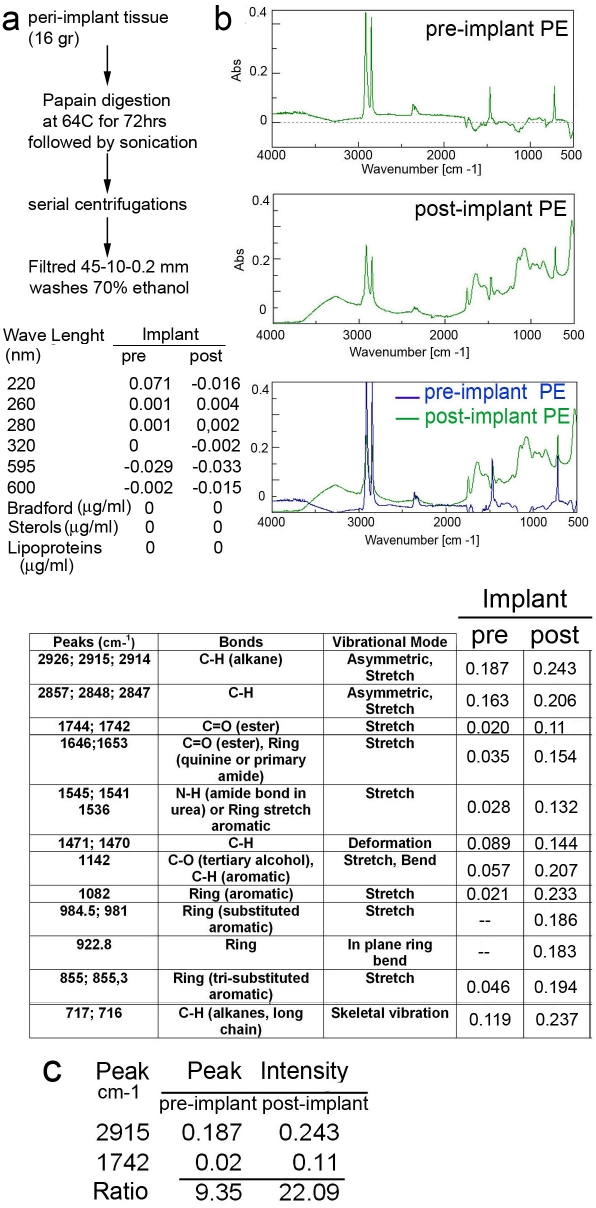
Modified alkane polymers purified from periprosthetic material increase their number of carbonyl groups. a) Purification scheme of post implant PE. Optical density of the preparation was measured at different relevant wavelengths at a PE concentration of 100 μg/ml. Bradford, lipid and sterol assays were also performed using100 ug/ml post-implant PE material. b) Fourier transformed infra-red spectroscopic (FTIR) analysis pre-implant (new polyethylene implant) and post-implant PE (polyethylene material purified from the site of osteolysis). The analysis was carried out between 4000 and 500 cm^−1^ wave number. b) Tabular representation of the prominent peaks (cm^−1^), and the predicted bonds with the relevant vibrational modes for pre-implant and post-implant PE. Integral intensities are given for each prominent peak. c) Total integral intensity of alkane and carbonyl groups in pre and post-implant material. Ratio between the two groups is shown.

**Figure 3:**
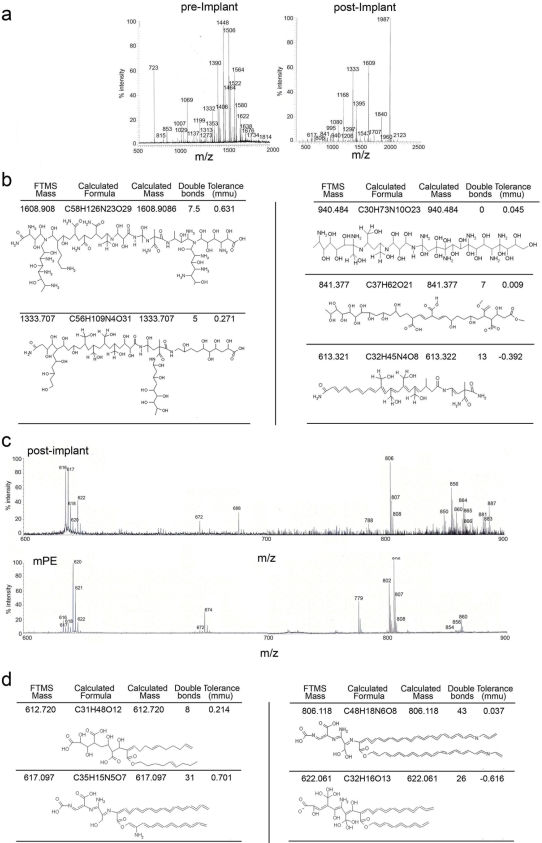
Mass spectroscopy analysis of n-alkane polymers purified from post implant PE indicates extensive oxidation. a) Fourier transformed FT/MS-MALDI analysis of pre and post implant PE polymers (molecular mass ranging between 400 and 2000). b) Predicted chemical composition for major peaks of post implant PE derived from FT-MS analysis depicted in a. c) MALDI-TOF scan (600–900 m/z) of PE purified from post-implant PE and mPE. d) Predicted chemical composition for major peaks of mPE and post implant PE depicted in c.

The presence of PE material at the site of inflammation raised the question of the molecular basis for alkane polymers recognition by the immune system. As initial analysis, a biophysical evaluation by fourier transformed infra-red spectroscopic (FTIR) [11] was performed on pre and post-implant material to determine possible alkane polymer modification that could explain the loss of its bio-inert properties (Figure 2). Analysis was performed on (i) pre-implant PE, corresponding to the actual biological implant before surgery, and (ii) post-implant PE, which was retrieved from the site of osteolysis at the time of revision surgery (Figure 2a and 2b). Post-implant material was purified according to scheme reported in Figure 2a. Lipid, sterols and protein analysis determined that the preparation was void of detectable contaminants as previously reported [8] (FTMS analysis and assignment of molecular formulas further confirm purity from lipids and protein contaminants (Figure 3a).

FTIR signature peaks at 2900–2800 cm^−1^ wave numbers showed the asymmetric stretch of the alkane backbone and a skeletal vibration of the same backbone was registered at 715 cm^−1^ for all the four samples. The alkane backbone also exhibited a predicted deformation at around 1470 m^−1^ in each case. Differences in spectra were noted between the pre and post implant PE material. A dramatic increase in the amount of carbonyl (carboxylic, ketonic, aldehydic and ester), amide and alcohol groups was observed in PE polymers prepared from post-implant material (Figure 2b). The increase in the amount of carbonyl groups (236% Figure 2c) is due to an “in situ” oxidative process. This oxidative process may be mediated by enzymes released from activated DC, MΦ and osteoclasts, or may occur in the endosomal compartment of local antigen presenting cells engorged with PE polymers (Figure 1g, 1h).

To further define the molecular composition of the alkane polymers retrieved from the post-implant material a Fourier transform ion cyclotron mass spectroscopy FT/MS in MALDI (matrix assisted laser desorption ionization) mode was performed. FT/MS is so far the most sophisticated technique to identify molecular composition since it combines the most advanced Ion Trap and Fourier Transform Ion Cyclotron Resonance technologies into a single instrument with unprecedented analytical power. Ultra-high resolution and sensitivity coupled with sub mass prediction accuracy allow determining elemental composition. Post-implant material was obtained as described in Figure 2b and short chain alkane polymers were further purified from the nano and micron size PE particles by centrifugation through a 10,000 membrane cut-off. Pre-implant material was similarly prepared. FTMS analysis indicated the presence of several small sized PE polymers in the post-implant material with a molecular mass of 400 to 2000 (Figure 3a). Differently from the pre-implant PE, where a clear polymer-associated envelope was observed, in the post implant material this signature was lost due to extensive oxidation. Chemical formulas were assigned for each molecular species observed in the post-implant PE. Assignment of each pick was consistent with an alkane structure back bone bearing several side chain modifications (Figure 3a). None of the assigned formulas could account for the presence of proteins, peptides, lipoproteins or lipopeptides further confirming the purity of the post-implant preparation. Most of the high molecular weight alkane polymers presented extensive addition of carboxyl groups (Figure 3b and Figure S1). In the lower molecular weight mass several polymers could be observed with a low degree of oxidation similar to the MS/MS profile, of mPE polymers (chemically synthesized PE polymers with few side chain modifications (hydroxyl and carboxyl functional groups) (Figure 3c and 3d and Figure S2). In both samples two major clusters of peaks in the 600 and 800 mass range could be observed (Figure 3c). Further analysis by TOF-TOF MALDI [12] fragmentation indicated an identical fragmentation pattern for both mPE and post implant PE. In both cases the 617 peak fragmented into 428 and 572 m/z, while the 806 peak fragmented into 617 (Figure S3). We therefore concluded that polymers of different sizes and amount of oxidation were present at the site of inflammation associated with a strong innate immune response.

**Figure 4:**
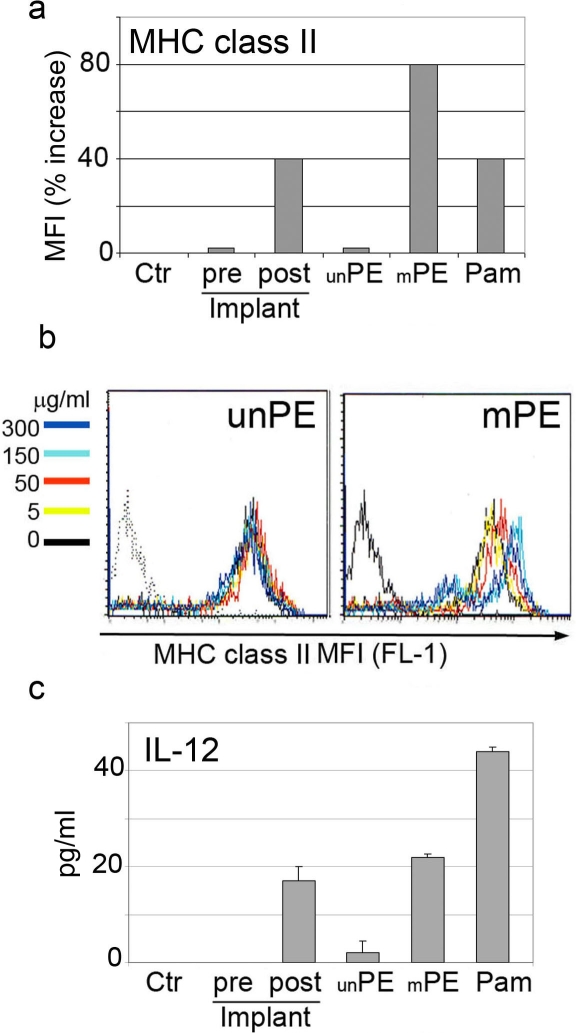
Modified alkane polymers induce DC activation and IL-12 secretion. a) FACS analysis of DC untreated or treated for 48 hours with unPE, mPE, pre and post implant alkane polymers. Surface staining was performed with antibodies to MHC class II molecules, Data are shown as increase in MFI as compared to the untreated control. b) Histograms overlay of MHC class II staining in DC untreated or treated with different concentrations of unPE and mPE. c) Elisa for IL-12 present in the supernatant of DC untreated or treated for 48 hours with unPE, mPE, pre and post implant alkane polymers (data are expressed in pg/ml).

The observation of modified PE material at the site of inflammation associated with cellular infiltrates prompted us to evaluate whether exposure to the modified polymers would activate antigen presenting cells. To test for this, we used pre-implant PE, post-implant PE, and chemically synthesized PE polymers without (unPE) or with side chain modification (hydroxyl and carboxyl functional groups) (mPE) matching several of the modification observed in the post-implant PE (Figure 3c). Dendritic cells (DC) cultured for 48 hours in the presence of 50 μg/ml of each compound were evaluated for surface MHC class II expression. An up-regulation of MHC class II molecules was observed in post-implant and mPE treated cells only, to level similar to what observed with the lipopeptide Pam2CSK4 (Figure 4a, 4b). DC activation was further confirmed by a significant increase in IL-12 secretion determined by ELISA (Figure 4c). We concluded that the hydroxyl and carboxyl modified mPE polymers and post-implant PE can interact with DC and initiate an inflammatory response.

**Figure 5:**
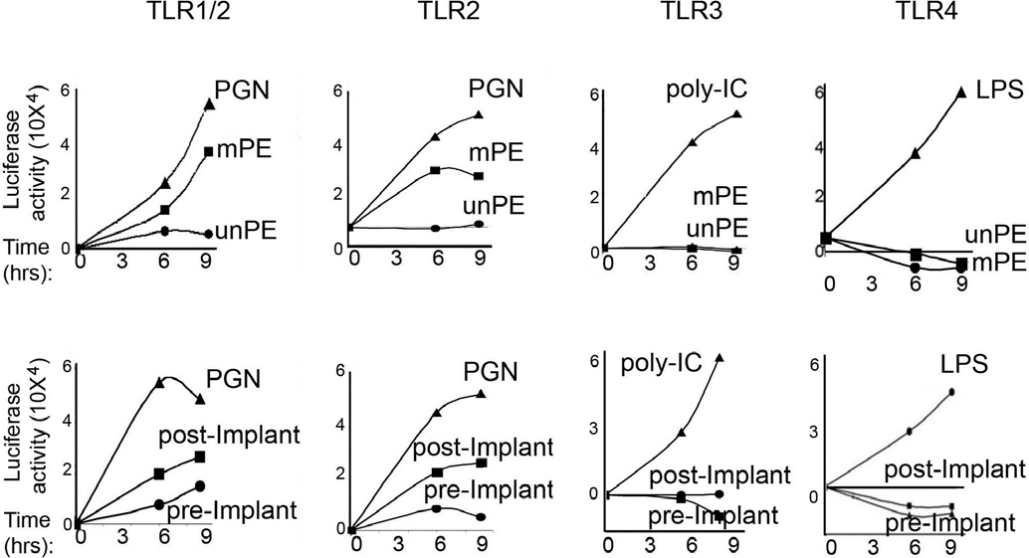
Modified alkane polymers induce activation of TLR-1 and TLR-2 signaling pathways. a) Luciferase activity expressed by human TLR1/2, TLR2, TLR3 and TLR4 stable HEK 3T3 transfectant (pNF-κB-LUC Stratagene). Cells were left untreated or treated for a different time period with 50 μg/ml of unPE, mPE or pre and post implant PE and respective positive controls; PGN(10 μg/ml) for TLR1/2 and TLR2, Poly (I∶C)(1 μg/ml) for TLR3 and LPS (10 μg/ml) for TLR4.

A strong and rapid initiation and activation of the innate immune response is generally achieved through engagement of members of the toll like receptor (TLR) family [13], [14]. Hence, we investigated further whether any of the TLRs were actually involved in the recognition of the mPE as well as post-implant modified alkane polymers. Human 3T3 HEK cell lines stably expressing TLR1/2, TLR2, TLR3 and TLR4 genes respectively were transfected with a plasmid encoding the luciferase reporter gene under the control of the NF-κΒ enhancer element. Thus, NF-κΒ activation by pro-inflammatory stimuli would, in turn, up-regulate luciferase production. Each TLR cell line was assayed for activation with different PE preparations, chemically synthesized (unPE and mPE) or alkane polymers prepared from the pre implant and post implant material [8]. Each assay also included a well known specific positive ligand for each respective TLR (Figure 5). Both mPE and post-implant preparations induced luciferase up-regulation in both TLR1/2 and TLR2 transfectants, but not in the TLR3 and TLR4 cell lines (Figure 5). Thus, we conclude that the carbonyl modified PE polymers cause a strong activation of the innate immune system and initiate an inflammatory cascade through the TLR1/2 receptor activation pathway.

**Figure 6:**
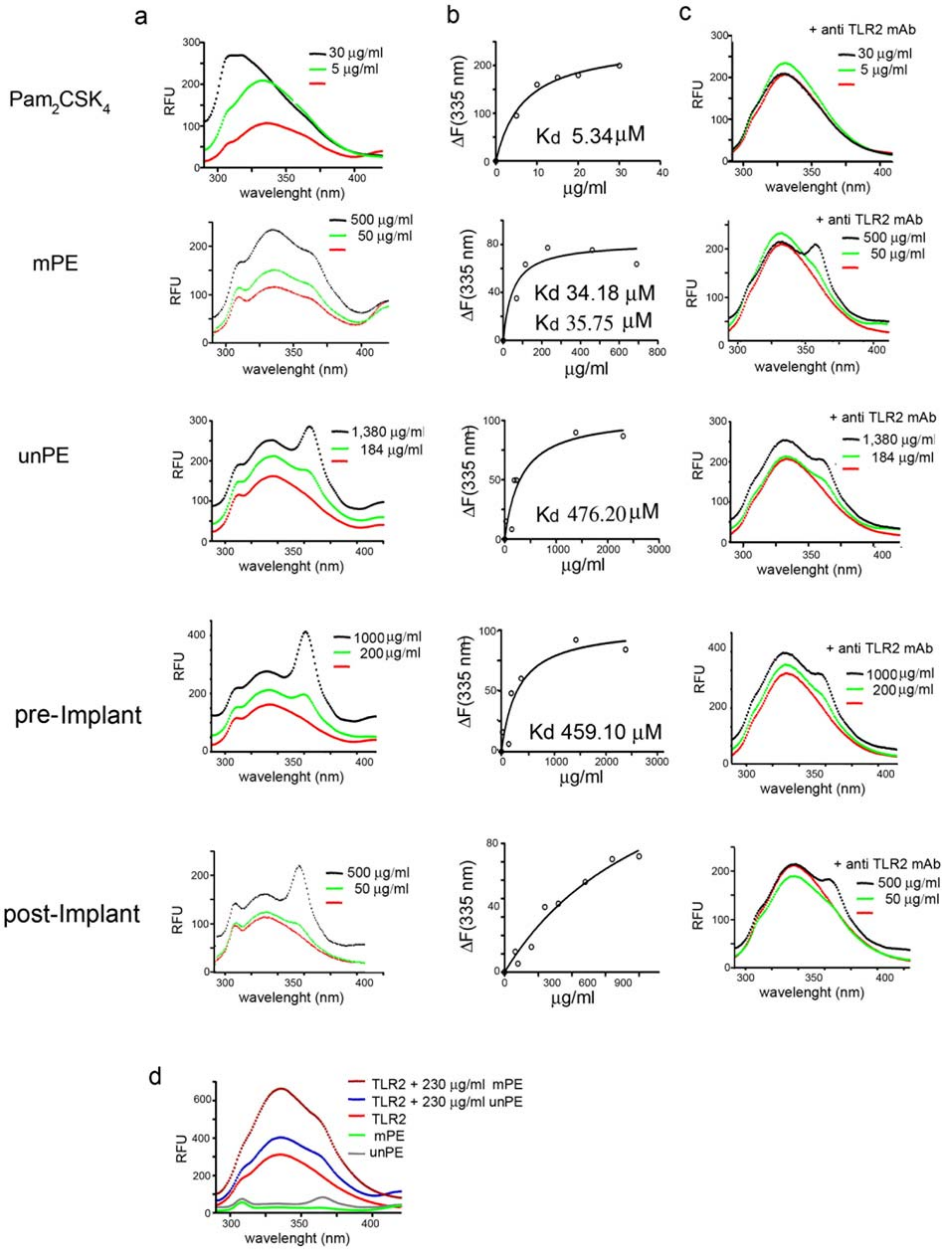
Direct binding of oxidized alkane polymers to soluble TLR-2 molecules. a) Left panels; fluorescence emission scans collected for free soluble TLR2 and TLR2 in complex with two different concentrations (exponential and plateau) of each analyzed polymer. Central panels; normalized fluorescence data (ΔF) for each concentration point as a function of the free ligand concentration. Right panels; fluorescence emission scans collected for free soluble TLR2 and TLR2 in complex with each analyzed polymer at two different concentrations (exponential and plateau) in presence of an anti TLR2 mAb known to block the TLR2 binding groove (stoichiometric ratio 2∶1 Ab to soluble TLR2 receptor). b) Comparison of the fluorescence emission scans collected for soluble TLR2, mPE and unPE.

To further analyze the TLR2 binding activity of modified alkane polymers a soluble form of human recombinant TLR2 (extracellular domain Glu 21-Leu 590) was utilized in a binding assay that monitored changes in fluorescence intensity (λ_excitation_ = 277 nm and λ_emission_ = 335 nm) of a tyrosine present in the TLR2 binding grove (Tyr 326) [15]. Changes in the binding grove environment, due to ligand occupancy would change the Tyr fluorescence intensity and the wavelength of its fluorescence emission. Soluble TLR-2 was incubated with increasing concentrations of each polyethylene derivatives; mPE (as a mixture of hydroxyl and ester bond alkane polymers), PE (unmodified alkane polymers) pre and post implant PE and the positive control, Pam2CSK4 lipopeptide (known to be a specific ligand for TLR-2) [15]. The emission scans (between 290 and 420 nm) were collected for each complex separately and the change in maximum fluorescence signal at 335 nm (due to tyrosinate ion) was used to generate the binding curves, after subtracting the contribution of the free protein (in the absence of any compounds). The normalized fluorescence data were fitted to a hyperbolic function using the software GraphPad Prism 4 (Figure 6).

The steady-state intrinsic fluorescence emission of TLR-2 strongly increased to ligand saturability upon the addition of mPE. Since mPE comprise a random mixture of hydroxyl and carboxyl alkanes (Figure S2) a binding Kd was calculated for both components. For hydroxyl and carboxyl modified alkanes the binding Kd was 34.18 μM and 35.75 μM respectively (Figure 6). Thus, the predicted Kd range for the modified alkane mixture was very close to the Pam2CSK4 positive control (Figure 6). Importantly, the alkane binding to soluble TLR-2 was inhibited following incubation with the monoclonal antibody anti human TLR2 (clone 383936 R&D Systems) which is known to prevent ligand access to the TLR2 binding groove [16]. On the other hand the affinity of binding for non-modified alkane polymers (unPE) and pre-implant material was much lower than the one reported for the oxidized ones (Figure 6). Post-implant PE, a wide mixture of non-oxidized and oxidized alkanes also showed a saturable binding even though the blend of different alkane species did not allow for Kd mesurement to be calculated. Nevertheless, the binding was completely blocked by addition of the TLR2 mAb (Figure 6). In general a positive correlation was observed between TLR2 binding affinity and amount of oxidation of the alkane polymeric structures (Figure 6b) in agreement with the luciferase assay data (Figure 5).

**Figure 7:**
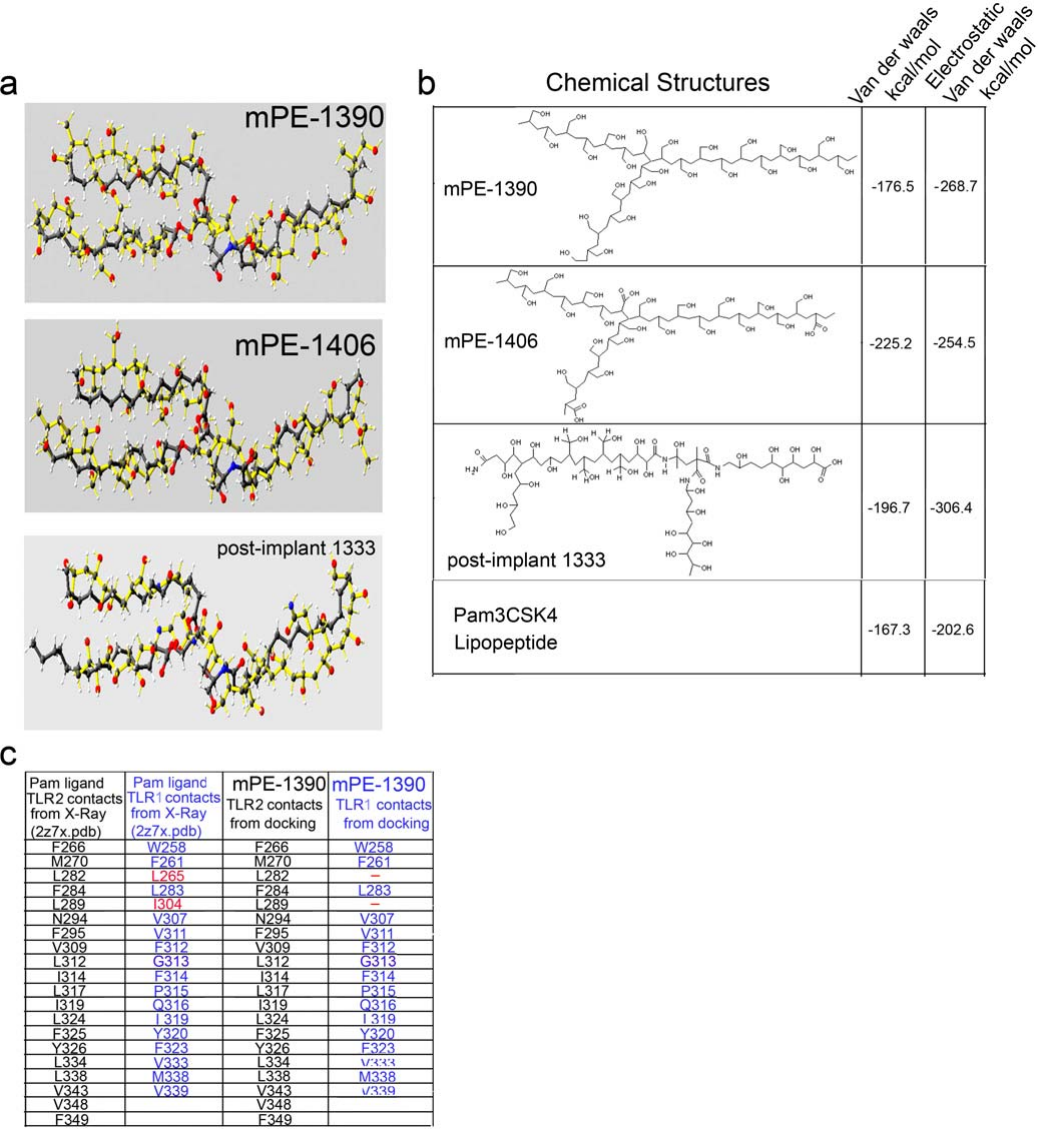
Mass spectroscopy analysis of n-alkane polymers predicted conformations fitting within the hydrophobic pocket of TLR-1/TLR-2 receptor. a) Docking and predicted conformations for the 1390 and 1406 mPE fragments and 1333 post implant PE fragment. b) Predicted relative free energies of interaction between the ligands reported in a and the TLR-1/TLR-2 receptor determined with the MM94FF force field. c) Amino-acids within the binding site of TLR-1/TLR-2 that are making contacts with the ligands Pam (from the original 2z7x.pdb structure) and with the mPE ligand (mPE-1390).

The crystal structure of TLR1/2 combined with an active bacterial ligand (Pam_3_CSK_4_) indicates that the CH2 backbone of the lipid ligand occupies the three hydrophobic active pockets [15]. Since FTIR analysis indicated that in the post-implant material alkane oxidation was the prevalent modification of the alkane groups we further characterize the interaction between the oxidized alkane polymers and TLR1/2 receptors. The structural fitness of; (**i**) a 24 repetitive units of an alcohol modified alkane polymer (1390 m/z), (**ii**) its oxidized form (1406 m/z), (**iii**) and an isomer from a post-implant polymer (1333 m/z) into the binding site of TLR-1/2 were evaluated. Molecular docking was performed using as template the 2z7x.pdb structure of the TLR-1/2 in complex with the tri-acylated lipopeptide Pam3CSk4 [15] (Figure 7). The mPE and post-implant polymers were designed to have an m/z ratio extrapolated from the FT/MS data (Figure 3a and Figure S2) and all the polymers had at least 16 carbons in their backbone based on published structural requirements for a TLR1/2 ligand [17]. The polymer structures were merged into the X-Ray structure 2z7x.pdb, such that each individual polymer was superimposed over the original ligand Pam_3_CSK_4_. The Pam ligand was then deleted to generate the TLR1/2-mPE complex (Figure 7a). The relative free energy of interaction between each of the new ligands and the TLR1/2 heterodimer was assessed using the MM94FF force field built-in the software SCULPT. Both the hydroxyl-modified alkane (mPE-1390) and the carboxyl form (mPE-1406) as well as the post implant 1333 were predicted to be very good fit based on the relative free energy of interaction as compared to the Pam_3_CSK_4_-lipopeptide control (Figure 7b). Thus, additional structural analysis of the interaction between mPE-1390 and TLR1/2 was performed by extracting the amino acids from the binding pocket of the receptor which made contacts with the ligand (Figure 7c). Altogether, the predicted relative free energy of binding, as determined by Vander waals and electrostatic interactions (kcal/mol), together with the analysis of amino acids contacts between the ligand and the receptor clearly indicated a strong probability for hydroxyl-modified alkane polymers to fit appropriately into the TLR1/2 binding groove.

## Discussion

Our study shows for the first time that organic synthetic polymers with an alkane subunit backbone and different carbonyl/amide side chain modifications can act as potent TLR2 and TLR1/2 activators.

It has been previously reported that extensive damage occurs in implanted akane polymeric structures in particular at sites of weight bearing. The process of PE breakdown generates nanometers and micrometers size particles, easily identifiable in the peri-implant tissue as well as in endosomal compartment of resident osteoclast and macrophages [3]–[10], [18], [19]. Herein, we also identify alkane polymers as additional products of PE breakdown. Interaction between PE particles/polymers and the host cells leads to oxidative changes by means of enzymatic, extracellular matrix-degrading activity of cells that have phagocytosed them or adhered to them. Under hyper-oxidative conditions and in presence of many enzymatic complexes, among which lipooxygenases, the free radicals could react fast with oxygen giving peroxide ROO• radicals and generate different alkane polymers with aldehyde, ketonic and hydroxyl groups as determined by FTIR, and FTMS (Figure 2 and 3). On the other hand the free polymeric radicals could re-react by cross linking each other and regenerate a saturated polymeric structure as well as formation of unsaturated structures with double bonds; both modifications were observed by FTMS (Figure 2 and 3). Altogether our data indicate that the PE breakdown and oxidation process released several carbonyl-modified alkane polymers with carbon chains of different length in the periprosthetic tissue.

Toll like receptors (TLR) are important pattern recognition receptors of the innate immune system. Among those TLR 2 is responsible for the functional recognition of lipoproteins/peptide constituent of the cell wall of bacteria [13], [14], [17], [20], [21]. TLR2 is different from other TLRs in that it is also activated by heterotypic interactions with TLR1 and TLR6. It was generally accepted that tryacylated lipopeptides are recognized by TLR1/TLR2, whereas dyacylated lipopeptides are recognized by TLR2/TLR6 [21]. Such dogma was recently challenged by reports that dyacylated lipopeptide could engage TLR2 in a TLR6 independent manner raising the possibility that TLR2 might be able to signal as homomers [20]. Recent analysis of the structural requirements for optimal TLR2 ligands was performed and indicated that two ester bond fatty acids with at least 12 carbon atoms were required to stimulate a cellular response [17]. In contrast, amide-bound fatty acids had no remarkable effect on TLR2 recognition even though they could determine the co-receptor usage (TLR1 vs. TLR6) [17].

Herein, a thorough biophysical analysis determined that the post implant PE retrieved at the site of inflammation encompass different carbon chain length alkane polymers modified by the addition of carboxyl groups. The mixture of the oxidized polymers were capable of activating TLR2 and TLR1/2 transfectant only few folds above the range of activation observed with the pre-implant material. This result has to be interpreted in light of the fact that several alkane species are present in the peri-implant tissue bearing different degrees of modification and carbon chain length, obviously not all of them compatible with TLR1/2 engagement. However, the requirement of alkane oxidation was further confirmed using chemically synthesized PE alkane with or without hydroxyl and carboxyl groups. Using this controlled in vitro system we could determine that carboxyl-modified alkanes were able to engage a soluble TLR2 with a 140 times greater binding affinity as compared to the non oxidized polymers. Molecular docking further confirmed that mPE polymers avidly fit into the hydrophobic groove of the TLR1/2 heterodimeric crystal structure.

Altogether our results show that the strong immune reaction following the break down of PE implant can be attributed to TLR1/2 engagement. This results can provide the molecular basis for the strong macrophages, osteoclasts and granulocytes activation observed at the site of the implant as well as after in vitro treatment with PE generated polymers [2]–[10], [18], [19], [22]–[29]. Important implications can be drawn for designing n-alkane/alkenes polymeric compounds with similar bio-performance but reduced immunogenecity.

## Materials and Methods

### Polyethylene

Ultra high molecular weight polyethylene unmodified (unPE) and carbonyl modified (mPE) were purchased from Sigma (cat# 429015 and # 434272 respectively). Both forms were prepared at 20 mg/ml in PBS and added in culture media at different amounts as reported in each figure legend. In some experiments a 10,000 MW cut off of PE preparations was utilized to test for the presence of shorter polymers. Post implant PE particles and polymers were purified from peri-prosthetic tissue removed at the time of the hip or knee revision surgery. All patients were diagnosed with loosening of the implant due to aseptic osteolysis and immune reaction to PE material. The scheme for PE purification is presented in Figure 2a as previously published [8].

### Immunohistochemistry

Tissue was formalin-fixed and paraffin embedded and cut on a microtome (4 um). Tissue slides were dried over night at room temperature, deparaffinize with xylene, cleared with graded ETOH (100%×2, 95%, 70%), and rinsed with ddH_2_O. Antigen retrieval was performed by microwave using Citrate buffer pH6.0 at 97o for 10 minutes. Slides were incubated with the following primary antibodies for 30 minutes: CD20 monoclonal mouse anti-human (mAb) (clone L26; dilution 1∶3000), CD3 polyclonal rabbit anti-human (#A0452; dilution 1∶200), CD68 mAb (clone PGM-1; dilution 1∶25) all from Dako Corp. (Carpinteria, CA). Antigen-antibody reaction was visualized using diaminobenzidine chromogen (DAB) applied for 7 minutes. Sections were counterstained in Hematoxylin for 30 seconds, cleared in 2% Glacial acetic acid for 30 seconds, rinsed in hot water then in 0.2% Ammonia Water for 10 seconds, rinsed in water, then dehydrated in ETOH and in Xylene before manual cover slipping. Samples were analyzed by light microscopy and polarized light microscopy to identified birefringent polyethylene particles (PE).

### Electron Microscopy

Tissue was fixed with a mixture of 2% paraformaldheyde and 4% PVP in phosphate buffer 0.2 M, pH 7.4 at 4°C and epon embedded. DCs untreated or treated with mPE for 48 hours were similarly fixed. Samples were processed for ultra-thin sectioning. Contrast was obtained with a mixture of 2% methylcellulose (SIGMA) and 0.4% uranyl acetate pH4 (EMS). Samples were viewed under a CM120 Philips electron microscope.

### Dendritic cells preparation and FACS analysis

Peripheral blood was obtained from the New York Blood Bank. The CD14+ monocyte population was separated using CD14 conjugated MicroBeads (Miltenyi Biotec). Purified cells were cultured in GM-CSF/IL4 (30 ng/ml plus 10 ng/ml) (RD Systems) for 5 to 6 days in DMEM (GIBCO, Grand Island, NY, USA). In some experiments DC were cultured in presence or absence of unPE, mPE and pre and post implant PE for 48 hours. DC were then washed with cold PBS, and labeled for 30 min on ice with saturating amounts of anti human HLA-DR (clone TU36) (BD Biosciences, Pharmingen, San Diego, CA) in staining buffer (PBS/0.1 % BSA/ 0.01% NaN3). The Cells were analyzed using a FACSCalibur flow cytometer and cellquest software program (BD Biosciences Mountain View, CA, USA).

### ELISA

DC were cultured in presence or absence of unPE, mPE and pre and post implant PE as described above. Culture supernatant was collected, filtered through a 20 μm filter and assayed for human IL-12 (Biosource (Invitrogen) according to the manufacturer instruction.

### Luciferase assay

The HEK 293/TLR clones (Invivogen) were used to determine TLR1/2, TLR2, TLR3 and TLR4 activation by PE. Each clone was transfected with the NF-KB cis- reporter enhancer (pNF-κβ-LUC, Stratagene) and an independent GFP containing using Fugene 6 transfection reagent (Roche). The expression of GFP was measured by FACS. Forty-eight hours post transfection the cells were treated with unPE, mPE, pre and post-implant-PE, as well as a positive controls; PGN (peptidoglycan from *Staphylococcus aureus*) at a concentration of 10 μg/ml l for TLR1/2 and TLR2, Poly (I∶C) (polyinosinic∶polycytidylic acid) was used at 1 μg/ml for TLR3 and LPS (lipopolysaccharide) at a concentration of 10 μg/ml for TLR4. All the controls were purchased from Invivogen. The luciferase readout was measured at different time points using the standard Luciferase reporter assay kit (Promega).

### Fourier Transform Infra-red spectroscopic analysis

Pre-implant PE and post-implant PE were analyzed by Fourier transformed infrared (FTIR) spectroscopy. All FTIR data was collected using JASCO 6100 Infrared Spectrometer equipped with a Golden Gate Attenuated Total Reflectance (ATR) cell. In order to prevent contamination between samples, the ATR window was cleaned with methanol prior to each analysis. The data were then analyzed using JASCO's spectra manager software (BioRad's Know it all software and Fiveash Data Management polymer database.

### Equilibrium titrations of polyethylene compounds into hrTLR-2

Binding of different polyethylene compounds and of lipopeptide control Pam2CSK-4 to hrTLR-2 was determined by monitoring changes in the receptor's intrinsic tyrosine fluorescence emission. The steady state fluorescence emission spectra were collected between 290 and 420 nm using λ_excitation_ = 277 nm for tyrosine, 5 nm bandwidth for both emission and excitation wavelengths and a response time of 0.5 seconds for each scan. Soluble TLR-2 was titrated with increasing concentrations of each polyethylene derivatives unPE, mPE, pre and post implant PE and the positive control, Pam2CSK4 lipopeptide (known to be a specific ligand for TLR-2 [15]. For each titration assay complexes between TLR-2 and each compound were preformed by incubating the protein (15 or 76 nM) with different compounds at different concentrations in 20 mM Tris-HCl with 200 mM NaCL, pH = 8.0 buffer at 4°C for 1 hour. Before reading their fluorescence emission scans between 290 and 420 nm the complexes were equilibrated at room temperature. The emission scans were collected for each complex separately and the change in maximum fluorescence signal at 335 nm (due to tyrosinate ions) was used to generate the binding curves, after subtracting the contribution of the free protein (in the absence of any compounds). The normalized fluorescence data were fitted to a hyperbolic function (one single binding site model) using the software GraphPad Prism 4. Similar experiments were conducted in presence of mouse anti human TLR2 mAb (clone 383936 R&D Systems) which is known to prevent ligand access to the TLR2 binding groove. The anti TLR-2 mAb was pre-incubated with the human recombinant TLR-2 on ice for 30 minutes at a stoichiometric ratio 2∶1 (Ab: soluble receptor). Each binding compounds; unPE, mPE, pre and post implant material was then added at different concentrations to the preformed complexes. Emission scans were collected as described above.

### Spectroscopic Analysis

Optical density of the pre- and the post-implant were determined at a concentration of 100 ugm/ml at the relevant wavelength after proper blanking of the instrument (BioRad smart spec 3000). The Bradford assay was performed at the identical concentration using the Biorad Braford assay kit (Biorad # 500006). Protein concentration was determined from the standard plot. The absence of lipids and the sterol were determined using the Libermann-Burchard test.

### Mass Spectroscopic Analysis

Mass spectroscopic analysis of all PEs samples were performed using MALDI (matrix assisted laser desorption/ionization) time of flight (TOF) and MS/MS MALDI-TOF/TOF technologies. Data were acquired using an ABI-4700 mass analyzer (Applied Biosystems, Framingham, MA). All spectra were collected in the reflector mode. Samples were prepared by mixing equal amounts of matrix (α-cyano-4-hydroxycinnamic acid from Sigma) and sample, each dissolved in 50% acetonitrile and 50% 0.1% trifluororacetic acid (TFA). The samples were loaded on a 192 well engraved stainless steel plate (ABI 433375). Calibration was updated before each acquisition using a standard peptide mixture according to instrument protocol. The PEs subjected to MS analysis were pre-filtered through Amicon Centriplus (YM-10,000: 10,000 Da cut-off). Only the filtrates were used for MS analysis.

### FT-MS

Mass Spectra were acquired on a Varian Fourier transform Mass Spectrometer (FTMS) (Lake Forest, CA) equipped with a MALDI source. The spectra were imported from Varian Doc Viewer and the analysis of chemical composition of each peak of interest was performed with the built-in software.

### Molecular docking

Molecular docking was carried out using SCULPT “Interactive 3D Structural and Electronic Analysis” software package provided by MDL Information systems, Inc (San Leandro, CA). The 2z7x.pdb structure of the TLR-1/TLR-2 heterodimers receptor in complex with the tri-acylated lipopeptide Pam_3_CSK_4_ was used as target while the new PE polymer ligands were derived from the original structure of the monomer (C3H6O)n predicted by the Fourier Transformed Mass Spectroscopy (FT-MS) data (supplementary information Sx). The new PEs were drawn in ISIS DRAW (MDL) and imported into SCULPT superimposing each structure over the original Pam_3_CSK_4_ ligand which was deleted such that the template TLR1/TLR2 had in its binding groove the new PEs. During each cycle of minimization the protein was freezed while the ligand was thawed using the corresponding functions from the SCULPT software. This rigid docking procedure ensured the flexible conformational search for the ligand within the binding site while the protein remained in its original conformation. The minimization procedures used both Van der Waals potential for finding the best sterical fit of the ligand into the binding groove and the electrostatic potential provided by the MM94FF built-in SCULPT to assess the relative free energy of binding between the ligand and the target protein.

## Supporting Information

Figure S1 Mass spectroscopy analysis of n-alkane polymers purified from post implant PE indicates extensive oxidation. a) Predicted chemical composition for major peaks of post implant PE derived from FT-MS analysis depicted in Figure 3a.
(0.68 MB TIF)

Figure S2 FTMS analysis of mPE. a) Fourier transformed FT/MS-MALDI analysis of carboxyl-modified mPE polymers.
(0.53 MB TIF)

Figure S3 MALDI parent ions 617 and 806 from PE and mPE have a similar fragmentation pattern. MALDI-TOF-TOF analysis of precursor ion 617 fragmentation into 428 and 572 for post implant PE and mPE and MALDI-TOF-TOF fragmentation of precursor ion 808 into 617 for both pPE and mPE.
(0.52 MB TIF)
